# Conflict in the EMS Workforce: An Analysis of an Open-Ended Survey Question Reveals a Complex Assemblage of Stress, Burnout, and Pandemic-Related Factors Influencing Well-Being

**DOI:** 10.3390/ijerph20105861

**Published:** 2023-05-18

**Authors:** Halia Melnyk, Gennaro Di Tosto, Jonathan Powell, Ashish R. Panchal, Ann Scheck McAlearney

**Affiliations:** 1 Center for the Advancement of Team Science, Analytics, and Systems Thinking in Health Services and Implementation Science Research (CATALYST), College of Medicine, Ohio State University, Columbus, OH 43202, USA; gennaro.ditosto@osumc.edu (G.D.T.); ashish.panchal@osumc.edu (A.R.P.); ann.mcalearney@osumc.edu (A.S.M.); 2 National Registry of Emergency Medical Technicians, Columbus, OH 43223, USA; jpowell@nremt.org; 3 Division of Epidemiology, College of Public Health, Ohio State University, Columbus, OH 43210, USA; 4 Department of Emergency Medicine, Wexner Medical Center, Ohio State University, Columbus, OH 43210, USA; 5 Department of Family and Community Medicine, College of Medicine, Ohio State University, Columbus, OH 43210, USA

**Keywords:** COVID-19 pandemic, workplace conflict, workplace stress, burnout, EMS clinician well-being, occupational health, future of work, systems thinking, complexity

## Abstract

Emergency Medical Services (EMS) clinicians provide patient care within a high-stakes, unpredictable, and complex work environment in which conflict is inevitable. Our objective was to explore the extent to which added stressors of the pandemic exacerbated EMS workplace conflict. We administered our survey to a sample of U.S. nationally certified EMS clinicians during the COVID-19 pandemic in April 2022. Out of 1881 respondents, 46% (*n* = 857) experienced conflict and 79% (*n* = 674) provided free-text descriptions of their experience. The responses were analyzed for themes using qualitative content analysis, and they were then sorted into codes using word unit sets. Code counts, frequencies, and rankings were tabulated, enabling quantitative comparisons of the codes. Of the fifteen codes to emerge, stress (a precursor of burnout) and burnout-related fatigue were the key factors contributing to EMS workplace conflict. We mapped our codes to a conceptual model guided by the National Academies of Sciences, Engineering, and Medicine (NASEM) report on using a systems approach to address clinician burnout and professional well-being to explore implications for addressing conflict within that framework. Factors attributed to conflict mapped to all levels of the NASEM model, lending empirical legitimacy to a broad systems approach to fostering worker well-being. Our findings lead us to propose that active surveillance (enhanced management information and feedback systems) of frontline clinicians’ experiences during public health emergencies could increase the effectiveness of regulations and policies across the healthcare system. Ideally, the contributions of the occupational health discipline would become a mainstay of a sustained response to promote ongoing worker well-being. The maintenance of a robust EMS workforce, and by extension the health professionals in its operational sphere, is unquestionably essential to our preparedness for the likelihood that pandemic threats may become more commonplace.

## 1. Introduction

Worker well-being refers to a person’s subjective experience of the quality of their work life [1]. It is a multi-dimensional and dynamic concept that involves the interplay of many factors whose causal relationships and feedback loops are complex [1]. Conflict among healthcare workers has been described as part of a cluster of organizational outcomes that include job satisfaction, stress, and burnout [2]. These outcomes are important to monitor given that low job satisfaction and high levels of conflict, stress, and burnout can negatively impact individuals’ personal health and work outcomes [2]. Workplace conflict typically refers to disagreements, differences, or incompatibility between an individual and their superiors, subordinates, or peers which can lead to stress, anxiety, and conflict escalation if not treated early [3]. While our knowledge of conflict experienced by healthcare workers during the pandemic is sparse, we know that burnout increased dramatically during the pandemic [4,5], despite having been at historically high levels prior to the pandemic [6].

Our understanding of worker burnout levels, however, primarily relates to physicians and nurses [5,6,7,8,9]. As first responders, Emergency Medical Services (EMS) clinicians encounter traumatic events that may place them at risk for adverse health and work-related outcomes [10]. It has been demonstrated that up to 69% of EMS clinicians lack the time to emotionally recover from traumatic events, a circumstance which may be associated with increased mental stress [10]. Stress that has not been successfully managed is a precursor to burnout, which is characterized by fatigue or exhaustion, negativity toward one’s job, and reduced professional efficacy [4,11]. Notably, in one pre-pandemic study, burnout was found to be prevalent amongst EMS clinicians [12]. While the connections between burnout and conflict are not well understood, it is likely that these job-related sequelae were exacerbated by the pandemic, particularly given that stress is a factor common to both phenomena. Additionally, the detrimental effects of burnout on occupational performance such as absenteeism, higher turnover intentions, and diminished job satisfaction, are also the same as the effects of persistent conflict [3,13]. The primary intent of our survey was to measure the prevalence of burnout and its correlates specifically among EMS clinicians during the pandemic. We included an open-ended question to explore EMS clinician experiences of conflict given the link between burnout and conflict in the literature.

Workplace conflict has been documented by multiple clinical disciplines across all levels of our modern healthcare delivery system [14]. Conflict is considered an inevitable aspect of work life [2]. Yet, despite EMS clinicians’ continuous exposure to a high stakes, unpredictable, complex care environment in which conflict is likely to erupt, the phenomenon of conflict amongst EMS clinicians is poorly understood [13]. The objective of our open-ended survey question was to explore the extent to which the added stressors of the pandemic exacerbated EMS workplace conflict. To date, this topic has not been formally reviewed. We first aimed to develop a rich contextual picture of the key drivers of conflict. We then mapped our findings to a conceptual model guided by the National Academies of Sciences, Engineering, and Medicine (NASEM) report on the use of a systems approach to clinician burnout and professional well-being to explore implications for addressing conflict within that same framework [6]. Viewing conflict through a systems framework, one comprised of self-organizing interactions among its many components and governed by feedback mechanisms, may provide stakeholders with a foundation for developing agile, multi-level interventions to improve professional well-being [6,15,16].

## 2. Materials and Methods

### 2.1. Study Design, Population, and Setting

This was a multi-method analysis of EMS clinicians’ free-text responses to a survey question concerning workplace conflict due to the COVID-19 pandemic. The primary objective of the survey was to assess burnout in EMS clinicians working in the U.S. during the pandemic, then compare those results to pre-pandemic levels. The overall survey asked questions concerning demographics, workforce characteristics, and pandemic-related workplace policies and resources. Burnout was measured using the Copenhagen Burnout Inventory (CBI) [17]. Stress was measured using the reduced Perceived Stress Scale (PSS4) [18]. Additionally, the survey included a closed-ended question about conflict, “Do you feel that COVID-19 has exacerbated conflicts with your co-workers over the past 12 months?”, followed by an open-ended question, “If yes, please describe the nature of the conflict(s)”. The open-ended question is the focus of this evaluation.

### 2.2. Data Collection

The electronic survey was distributed during April 2022 to a simple random sample of 19,497 nationally certified, civilian, EMS clinicians aged 18–85 years who were in the National Registry of Emergency Medical Technicians database. The National Registry provides initial EMS certification (Emergency Medical Technician and National Registry Paramedic, Columbus, OH, USA) in more than 95% of U.S. states and territories. The study received expedited approval from The Ohio State University Institutional Review Board and the American Institutes for Research in the Behavioral Sciences Institutional Review Board.

### 2.3. Analysis

We applied a multi-method approach to analyze and interpret the responses to the open-ended question asking about the nature of conflict. Responses were first analyzed qualitatively using content analysis [19] to inductively arrive at non-hierarchical codes that represent themes that emerged from the type of conflicts experienced by EMS clinicians. HM drafted a preliminary coding dictionary from an initial review of the responses. HM and ASM iteratively refined codes contained in the dictionary by combining codes with similar underlying concepts and excluding codes contained in fewer than ten responses. Trustworthiness and credibility [20,21] were ensured by documentation during the assignment of codes and consultation with field experts ARP (EMS and EM physician, EMS medical director, Baltimore, MD, USA) and JP (Nationally Registered Paramedic, Columbus, OH, USA). Responses to the open-ended question were stored in an Excel database (v2211, Redmond, WA, USA).

Next, the responses contained in each code were analyzed to identify a word unit set (i.e., words, word stems, and words phrases) unique to that code. Excel was used to programmatically sort responses into the codes based on the content of their word unit sets. Analytical rigor [22] was ensured through checking responses for consistency with code definitions and by constant comparison of codes with their word unit sets. For example, if a response met multiple code definitions, it was cross-checked with each code. If a response met a code definition but had not been coded as such, the word set was expanded. Conversely, if a response was included in a code, but did not meet its definition the word set was narrowed to be more exclusive. Occasionally, responses that remained incorrectly coded were either forcibly included or excluded from a given code definition. Additionally, word unit sets were expanded to include their synonyms.

A pivot table was used to inspect the frequency of a code’s occurrence within the total sample of responses. The pivot table also facilitated the exploration of the density of codes found within any given response, which enabled the creation of co-occurrence matrices to display the coded data across multiple EMS descriptions of conflict.

Last, codes were considered within the context of the NASEM conceptual framework of burnout as a complex, multi-factorial problem for which an evidence base for system interventions is urgently needed (i.e., approaches that address factors found at the individual, institutional, and societal levels) [6]. Our presentation of findings follows the standards for reporting qualitative research (SRQR) guidelines [23].

### 2.4. Statistical Analysis

Descriptive statistics for the demographic and professional characteristics of the sample were performed. The burnout and stress measure scores were computed.

## 3. Results

The overall survey had a 10% response rate (N = 1882). The major finding was that the unweighted prevalence of EMS clinician burnout increased for all dimensions of the CBI from 2015 to 2022 [24]. Interestingly, in the current 2022 survey in which the conflict question was first posed, EMS clinicians who responded to the conflict question (*n* = 1686) also reported significantly higher levels of burnout and stress across all dimensions of the CBI and PSS4 instruments (Table 1, burnout and stress measures). The demographic and professional characteristics of those who answered “Yes” to experiencing workplace conflict (*n* = 857) were similar to those who answered “No” (*n* = 829). The only significant differences related to the fact that those reporting conflict had higher certification levels and longer shift lengths (Table 1, professional characteristics). Of those who reported conflict (*n* = 857), 79% provided free-text responses describing the nature of the conflict.

### 3.1. Variety, Frequency, and Density of Codes

EMS professionals attributed the nature and source of exacerbated conflict to a variety of factors that fell into 15 codes. These codes listed in order of frequency are as follows: (1) everyone’s on edge; (2) vaccine, mask, mandate; (3) different beliefs, opinions; (4) staffing difficulties, overwork; (5) burnout spectrum; (6) politics; (7) management issues; (8) policy, protocol issues; (9) COVID-19 magnification; (10) COVID-19 denial; (11) systems issues; (12) lack of trust, dishonesty; (13) rights infringement; (14) hostile, toxic workplace; (15) COVID-19 conspiracy (Table 2). In all, these codes represented 88% (*n* = 592) of the total number of free-text responses (*n* = 674).

The EMS responses describing conflict in the workplace during the pandemic ranged in length from one word or word phrase to a few sentences, and occasionally as much as a full paragraph. Six percent (*n* = 36) of the responses contained one word, the median number of words in a response was nine, and the maximum number of words in a response was one hundred forty-six. Referring to Table S1, the word unit set for each code ranged from as few as four words (i.e., see code: vaccine, mask, mandate) to as many as forty-seven words (i.e., see code: staffing difficulties, overwork) (Table S1). The maximum number of codes contained in a response was seven.

### 3.2. Complexity of Codes Contained in a Response

Overall, our findings revealed a high level of complexity in EMS respondents’ descriptions. This complexity was demonstrated by the frequent presence of multiple codes in any given response. For example, the codes “everyone’s on edge” and “burnout spectrum” appeared together in 27 responses (Table 2). Of those 27 responses, 18 included additional codes. In sum, the density of codes that may be found within any given free-text response in our survey suggests that EMS professionals perceive that multiple factors are complicit in exacerbating workplace conflict during the pandemic.

### 3.3. Association of Conflict with Burnout and Its Adverse Professional Outcomes

As noted earlier, burnout is a syndrome resulting from chronic workplace stress, and one of its hallmark characteristics is fatigue [6,11]. Not surprisingly, we found that stress and fatigue were also sources of conflict. The most frequently occurring code to emerge in our analysis, “everyone’s on edge” (*n* = 234), for example, attributed the source of exacerbated co-worker conflict to an organizational climate that was emblematic of a stressful work environment (i.e., people were easily irritated, frustrated, and quick to lose patience or become angry). In keeping with our topic relating to the link between conflict and burnout, the fifth most frequently occurring code to emerge was a range of fatigue complaints. These complaints were coded as “burnout spectrum” (i.e., people were tired, weary, fatigued, exhausted, burned out).

The range of fatigue complaints comprising the code “burnout spectrum” is consistent with the NASEM report that burnout exists on a continuum that varies in severity and presents differently across individuals [6]. Severe burnout has been associated with adverse professional outcomes among clinicians in general [6]. It can be inferred that EMS responses containing compound word descriptions such as “exhaustion, emotionally spent”, “across the board burnout”, and “extreme burn out” exist on the more severe end of the burnout spectrum. Moreover, the adverse outcomes of burnout described in the NASEM report (i.e., increased work effort, turnover, absenteeism; impaired job performance, decreased quality of and poor attitudes toward patient care) [6] were evident in the EMS responses containing the “burnout spectrum” code and often co-occurred with other conflict codes (refer to Table 3).

### 3.4. Mapping Conflict to the NASEM Framework of Burnout as a Complex Systems-Level Problem

**Figure 1:**
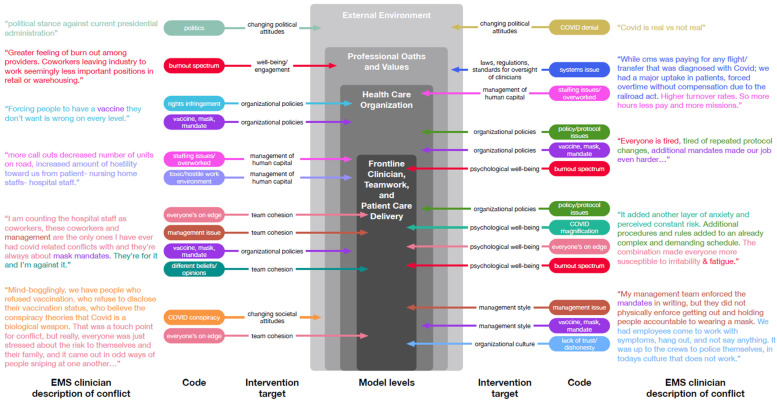
Codes Mapped to a Systems Model of Clinician Burnout and Professional Well-Being (guided by NASEM)^6^. **Legend** (from outside to inside): EMS clinician description of conflict: Direct quotes from EMS clinician responses about sources of conflict. Code: Fifteen codes emerged as sources of conflict. Many responses contained co-occurring codes. Intervention target: Components of the system that might be good targets for interventions. Model levels: Successful interventions would address the interactions and interdependencies between levels.

Mapping the variety of codes that emerged from our analysis to their corresponding component within the system levels of the NASEM framework further supported the link between conflict and burnout (Figure 1). By referring to a singular response depicted in Figure 1, for example, we found that it contained four codes (in italics):

“[COVID-19] added another layer of anxiety and perceived constant risk (*COVID magnification*). Additional procedures and rules added to an already complex and demanding schedule (*Policy*, *protocol issues*). The combination made everyone more susceptible to irritability (*Everyone’s on edge*) & fatigue (*Burnout spectrum*)”.

In the response above, the codes “COVID magnification”, “Everyone’s on edge”, and “Burnout spectrum” map as factors that directly relate to the psychological well-being of the frontline clinician (i.e., the inner core of the system model). The code “Policy, protocol issues” maps as an organizational factor (the second rung of the model), indirectly leading to stress and burnout in the frontline clinician. Our code mapping demonstrates that EMS professionals, on both an individual and group basis, attributed the source of exacerbated workplace conflict to a wide range of factors that are distributed across multiple levels of the system in which they provide care. These factors taken together potentiated the likelihood of conflict during the pandemic.

## 4. Discussion

This is the first study, to our knowledge, to present a relationship between increased conflict amongst EMS clinicians in the workplace and the phenomenon of burnout. Workplace conflict for clinicians can be damaging to their personal health and well-being. It may also have detrimental effects on work climate, professional performance, healthcare costs, and patient outcomes [13]; however, there are few studies addressing EMS insider insights into the drivers of conflict. Our multi-method analysis of free-text data from a national sample of EMS clinicians identified a variety of factors that exacerbated workplace conflict during the pandemic. Increased clinician stress and their experience along the “burnout spectrum” of fatigue/exhaustion are two key factors contributing to conflict in our study. Stress and fatigue are also attributes of burnout [6]. This finding is consistent with Wright (2011), and adds to the evidence that workplace conflict and burnout are interrelated [2]. Additionally, our research highlights that pandemic-specific stressors played a large role in exacerbating conflict. The leading stressors include vaccine and mask mandates; differences in beliefs and opinions; and staffing difficulties and/or overwork.

Conflict and burnout impact professional performance similarly. These shared effects include (1) increased work effort, turnover, absenteeism; (2) impaired job performance; and (3) decreased quality of care and poor attitudes toward patients [6,13]. The pre-pandemic NASEM report on the negative consequences of burnout for clinicians and its spillover effect on the performance of the U.S. healthcare system was prescient. Our findings add to the growing body of knowledge relating to the exacerbation of work-related stress and burnout amongst clinicians during the pandemic [4,7,8,9,25]. The findings also fill a gap in our understanding of the nature of conflict amongst EMS clinicians, which has few precedents in the literature [13].

The adverse effects of conflict on physical and mental health are also similar to those of burnout. These effects are cardiovascular disease, hypercholesterolemia, heart disease, and depression [6,13]. Given the adverse health effects of burnout, many European countries have designated burnout a medical diagnosis [6]. As such, workers with more severe cases of burnout may avail themselves of occupational resources and paid leave [6]. In North America, however, burnout is considered a work-related syndrome, and is not amenable to a medical diagnostic solution [6]. In the North American context, adding the phenomenon of conflict to burnout would emphasize the complexity of interrelationships among work environment factors that may contribute to our understanding of unhealthy occupational syndromes. The enriched framework suggested in this study is easily reconcilable with the Total Worker Health paradigm of the U.S. National Institute of Occupational Safety and Health. This paradigm includes interactions with co-workers as a job-related factor impacting worker well-being [26]. A national effort that deploys more intellectual and material resources to the occupational health clinical workforce could address the adverse consequences of these factors as presented in our study.

The novelty of this analysis is that it highlights the complexity and interplay of the multitude of factors that actually influenced EMS clinician well-being during the pandemic. The scope and complexity of our findings on conflict, especially as they dovetail with the NASEM conceptual framework of burnout, highlight the magnitude of the pandemic’s effect on worker well-being. Recognizing workplace conflict through a systems lens requires that we consider more than individual or mental health interventions in order to mitigate the problems of conflict and burnout. Mainly, the NASEM suggests encouraging opportunities for public and private partnerships among a broad array of stakeholders to engage in research on clinician professional well-being [6]. Governmental bodies, in particular those engaged in legislation relating to EMS and their sphere of operations, may wish to support research to mitigate clinician burnout in order to ensure a vital workforce. In sum, the NASEM imperative to address clinician burnout takes on heightened importance if we are to maintain a robust EMS workforce capable of fully responding to public health crises.

### Limitations

Our analysis and interpretation of EMS responses support the existence of a relationship between increased workplace conflict and burnout during the pandemic. These findings, however, while drawn from an EMS national registry of certified clinicians, may not represent all state-licensed, non-nationally certified EMS professionals.

In general, free-text responses to open-ended questions present certain analytical and interpretive limitations. A primary concern is that because a free-text response may consist of only a word or a sentence, it may produce results lacking in contextual and conceptual depth [27]. This concern was mitigated in our study by the large sample size and the broad range of response lengths noted earlier. Additionally, the use of a combination of code-based (qualitative) and word-based (quantitative) methodological approaches for the data analysis [28] ensured that the coding frames provided good coverage of the data, including both sparse and rich response types. This method also enhanced analytic rigor.

Another limitation of free-text responses is that quantitative closed-ended questions indicate the “legitimate agenda” for open-ended questions [27]. In effect, the free-text response to the open-ended question may be biased by the coloring and content of the quantitative questions. With regard to that concern, we reviewed the position of the open-ended question in the survey relative to the quantitative questions preceding it. The open-ended question in our survey was positioned after the CBI measure of burnout and several other questions related to COVID-19 workplace policies and vaccination/booster status. This positioning, therefore, may have influenced the EMS free-text responses toward the inclusion of factors presented in these earlier questions. However, burnout, workplace policies, and vaccination status did not emerge as the lead codes describing the nature of the conflict. In fact, the lead code was “Everyone’s on edge” (refer to Table 1). Given the wide variety of codes (15 code types) demonstrating different dimensions of EMS worker experiences, it is unlikely that question order bias grossly influenced our findings.

The inductive approach that we used to create our codes lent itself to a flat coding frame, in which all the codes are of the same level of specificity and importance (https://getthematic.com (accessed on 5 February 2023)) [29]. One drawback of a flat coding scheme, as opposed to a hierarchical one, is that less frequently occurring themes are either not captured or their codes are dropped from the final scheme [29]. Despite this limitation, 88% of all free-text responses were assigned a code, indicating that the size and coverage of the coding frame were robust (the codes covered many relevant survey responses).

A final limitation often noted about the survey format is that it does not allow for immediate follow-up questions to improve understanding. Interestingly, this limitation may be offset by certain strengths. Namely, open-ended survey questions may promote a more concise “list” form, while at the same time giving respondents the opportunity to “vent” or explain themselves in short narrative form [28]. The types of responses provided by EMS workers in our survey (e.g., use of epithets, expressions of disgruntlement, and other highly charged statements) suggest that many felt free to speak their minds in a fashion that may not have emerged with interview or focus group data collection.

## 5. Conclusions

The national scope of our sample and the richness of the EMS clinicians’ descriptive responses with regard to the sources of conflict should compel a policy response. The findings of our study as depicted in Figure 1 reveal that the national, political, and social climate; the actions of government; and local organizational policies all had a ripple effect on the ability of frontline clinicians to respond to crisis. Figure 1 also exposes the failures of the system at the intersection of EMS spheres of operation; the broken discourse between nursing homes, hospitals, and communities; as well as the deleterious impact of the irreconcilability of historical health-related legislation and the requirements of emerging pandemic regulations. The work capacity and morale of patient-facing professionals were stretched beyond the breaking point. Moreover, Figure 1 lays bare that the scope of the problem was beyond the capabilities of frontline managers to address solely via internal administrative means. As such, the application of systems thinking to our findings suggests that the active surveillance of clinician experiences during times of public health emergencies such as COVID-19 may provide real-time data vital to designing agile interventions to address heightened occupational stress. This approach may prove its worth many times over in the event that healthcare crises increase in likelihood [30,31]. It must also be noted that EMS clinicians would likely support these efforts due to their comments that receiving feedback about patient care is essential to promote optimal job performance [32,33,34]. In effect, continuous communication feedback loops are the change driver sine qua non in any complex adaptive system [15]. Information gathered from the active surveillance of frontline EMS teams could be profitably shared among clinicians, administrators, and governing bodies [35,36] to improve the quality of regulations and policies enacted during public health emergencies.

The pandemic has underscored the necessity of strengthening the global public health infrastructure in which clinician well-being across all practice settings has emerged as a critical, complex issue [37,38,39,40]. Readers will note, for example, that nurses and ambulance drivers in the U.K. National Health Service have engaged in costly and damaging strikes over work conditions [41]. In the U.S., pharmacists working at national drug company chains have reported record stress levels [42]. Stress and burnout are attributed as the reason for the turnover of roughly 100,000 nurses in the U.S. workforce, with more than six-times that number predicted to leave the profession within the next few years [43]. Our findings, while specific to EMS, are emblematic of the pandemic-strained healthcare system.

Firsthand frontline accounts from our healthcare workforce provide us with insights into what has been described by Navuluri et al. (2023) as “distressed work.” This work phenomenon is deeper and more pervasive than the stressful environment that many clinicians encounter on a daily basis [25]. The concept necessarily captures a chronic and profound imbalance vis-a-vis one’s vocational calling and the distress engendered by multiple pandemic-related work space rearrangements (e.g., as a result of infectious disease isolation protocols and mandates) and difficult co-worker, patient, and family interactions [25]. The emergence of distressed work is instrumental to healthcare provider decisions to exit the profession while simultaneously acting as a disincentive to new entrants into the field. We are obliged to take action that helps the clinical workforce to successfully navigate this changed landscape. We propose, as revealed by our choice of methodology, that occupational health policies be responsive to healthcare workers’ real-time, felt experiences of the system that is so critical for our future health.

## Figures and Tables

**Table 1:** Differences in Characteristics of Emergency Medical Services Clinicians With/Without the Experience of Workplace Conflict During the COVID-19 Pandemic.

Characteristic	Overall *n* = 1686 ^1^	No Conflict *n* = 829 ^1^	Yes Conflict *n* = 857 ^1^	*p*-Value ^2^
**Demographic**				
**Sex**				0.98
Female	448 (27%)	220 (27%)	228 (27%)	
Male	1224 (73%)	602 (73%)	622 (73%)	
Unknown	14	7	7	
**Age**	42 (13)	42 (13)	41 (12)	0.24
**Race/Ethnicity**				0.44
White, Non-Hispanic	1410 (87%)	692 (86%)	718 (88%)	
All others	208 (13%)	108 (14%)	100 (12%)	
Unknown	68	29	39	
**Education**				0.54
High school or less	189 (11%)	89 (11%)	100 (12%)	
Some college	523 (31%)	248 (30%)	275 (32%)	
Associate’s degree	321 (19%)	157 (19%)	164 (19%)	
Bachelor’s degree or higher	653 (39%)	335 (40%)	318 (37%)	
**Employment status**				0.27
Part time	154 (11%)	80 (12%)	74 (10%)	
Full time	1049 (77%)	493 (75%)	556 (78%)	
Volunteer	168 (12%)	88 (13%)	80 (11%)	
Unknown	315	168	147	
**Professional**				
**Certification level**				0.007
Emergency Medical Technician (EMT)	551 (33%)	297 (36%)	254 (30%)	
National Registry Paramedic (NRP)	1135 (67%)	532 (64%)	603 (70%)	
**Agency type**				0.96
Fire department	489 (35%)	233 (35%)	256 (36%)	
Private	348 (25%)	166 (25%)	182 (25%)	
Government, non-fire department	247 (18%)	120 (18%)	127 (18%)	
Hospital	184 (13%)	87 (13%)	97 (13%)	
Other	121 (9%)	62 (9%)	59 (8%)	
Unknown	297	161	136	
**Type of service provided at main EMS employer**				0.84
911	535 (72%)	261 (74%)	274 (71%)	
Medical transport	37 (5%)	15 (4%)	22 (6%)	
911 and medical transport	128 (17%)	58 (16%)	70 (18%)	
Clinical services	0 (0%)	0 (0%)	0 (0%)	
Mobile-integrated healthcare community paramedicine	9 (1%)	4 (1%)	5 (1%)	
Other	34 (4.6%)	17 (4.8%)	17 (4.4%)	
Unknown	943	474	469	
**Years of experience**				0.17
Less than 5 years	361 (21%)	193 (23%)	168 (20%)	
5 to 15 years	634 (38%)	301 (36%)	333 (39%)	
More than 15 years	691 (41%)	335 (40%)	356 (42%)	
**Shift length**				0.03
Less than 12 h	210 (12%)	101 (12%)	109 (13%)	
12 h	528 (31%)	258 (31%)	270 (32%)	
24 h or more	812 (48%)	389 (47%)	423 (50%)	
Other	131 (8%)	81 (10%)	50 (6%)	
Unknown	5	0	5	
**Burnout ^3^ and Stress ^4^**				
**Personal burnout**	50 (23)	45 (23)	55 (22)	<0.001
Unknown	19	8	11	
**Work-related burnout**	49 (20)	44 (20)	53 (19)	<0.001
Unknown	24	13	11	
**Patient-related burnout**	29 (23)	25 (22)	34 (24)	<0.001
Unknown	203	96	107	
**Perceived stress, overall**	6 (3)	5 (3)	6 (3)	<0.001
Unknown	24	11	13	

^1^*n* (%); mean (SD); ^2^ Pearson’s Chi-squared test; Wilcoxon rank sum test; Fisher’s exact test; ^3^ Copenhagen Burnout Inventory; ^4^ Perceived Stress Scale 4.

**Table 2:** Code Variety, Frequency, and Complexity in EMS Clinician Descriptions of Workplace Conflict.

**All Codes**															
**Column:**	**Code 1**	**Code 2**	**Code 3**	**Code 4**	**Code 5**	**Code 6**	**Code 7**	**Code 8**	**Code 9**	**Code 10**	**Code 11**	**Code 12**	**Code 13**	**Code 14**	**Code 15**
**Row:** **Code Count**	**Everyone Is on Edge**	**Vaccine, Mask, Mandate**	**Different Beliefs, Opinions**	**Staffing Difficulty, Overwork**	**Burnout Spectrum**	**Politics**	**Management Issue**	**Policy, Protocol Issue**	**COVID-19 Magnification**	**COVID-19 Denial**	**System Issue**	**Lack of Trust, Dishonest**	**Rights Infringement**	**Toxic, Hostile Workplace**	**COVID-19 Conspiracy**
1	72	49	38	26	23	28	9	3	3	5	2	3	2	6	2
2	84	89	45	36	27	16	19	13	14	11	11	6	8	5	6
3	48	40	25	31	19	15	21	9	12	9	8	7	5	4	2
4	20	15	13	12	6	7	7	6	4	5	4	6	3	2	2
5	6	4	5	2	1	4	4	4	0	2	3	3	2	0	0
6	3	2	2	2	1	2	2	0	1	0	2	1	0	0	0
7	1	2	1	2	1	0	2	2	0	0	1	0	1	1	0
**Grand Total**	234	201	129	111	78	72	64	37	34	32	31	26	21	18	12
Note. Row 1 = Number of times a code appears by itself in a response; Row 2 = Number of times a code appears with 1 other code; Row 3 = Number of times a code appears with 2 other codes; Row 4 = Number of times a code appears with 3 other codes; Row 5 = Number of times a code appears with 4 other codes; Row 6 = Number of times a code appears with 5 other codes; Row 7 = Number of times a code appears with 6 other codes.															
**Co-occurrence Matrix for “Everyone’s on Edge” and “Burnout Spectrum” Codes**															
**Column:**	**Code 1**	**Code 2**	**Code 3**	**Code 4**	**Code 5**	**Code 6**	**Code 7**	**Code 8**	**Code 9**	**Code 10**	**Code 11**	**Code 12**	**Code 13**	**Code 14**	**Code 15**
**Row:** **Code Count**	**Everyone Is on Edge**	**Vaccine, Mask, Mandate**	**Different Beliefs, Opinions**	**Staffing Difficulty, Overwork**	**Burnout Spectrum**	**Politics**	**Management Issue**	**Policy, Protocol Issue**	**COVID-19 Magnification**	**COVID-19 Denial**	**System Issue**	**Lack of Trust, Dishonest**	**Rights Infringement**	**Toxic, Hostile Workplace**	**COVID-19 Conspiracy**
2	9	0	0	0	9	0	0	0	0	0	0	0	0	0	0
3	12	1	0	4	12	0	1	0	5	0	0	0	0	0	1
4	4	0	1	2	4	1	1	1	1	0	1	0	0	0	0
5	1	1	0	0	1	1	0	1	0	0	0	0	0	0	0
6	1	1	0	0	1	0	1	0	1	0	1	0	0	0	0
**Grand Total**	27	3	1	6	27	2	3	2	7	0	2	0	0	0	1
Note. Row 2 = Number of times “Everyone’s on edge” and “Burnout spectrum” codes appear together in a response; Row 3 = Number of times they appear together with 1 other code; Row 4 = Number of times they appear together with 2 other codes; Row 5 = Number of times they appear together with 3 other codes; Row 6 = Number of times they appear together with 4 other codes. Grand Total = Number of times that a code was included with “Everyone’s on edge” and “Burnout spectrum” in a response.															

**Table 3:** EMS Clinician Conflict Responses Coded with “Burnout Spectrum” that Illustrate Adverse Effects on Professional Performance Described in the NASEM Report on Burnout.

Adverse Outcome	Illustrative EMS Clinician Response	Co-Occurring Codes
▪ Increased work effort ▪ Impaired job performance ▪ Decreased productivity	“COVID-19 had a direct impact on the mental and physical fatigue of every one of my co-workers. there were several times that an entire shift would be out with the virus, which left the other crews responsible for picking up extra shifts to cover in an already exhausting environment. i work in a very rural area and the local hospital is not equipped to handle a severely sick COVID pt, so during the height of the pandemic we were transferring a lot of patients to other hospitals. the nearest appropriate hospital is 70 miles away, and sometimes we would spend 6 to 8 h waiting for a bed at that hospital. many many shifts i would not have a moment of rest out of a 48 h shift”.	▪ Burnout spectrum ▪ Staffing difficulty, overwork ▪ Systems issues
▪ Increased work effort ▪ Disengagement from job	“our company is severly [SIC] understaffed with no real attempts to try to make it so people. would want to come there to work due to that it’s always the same people running and we are tired and exhausted and taking it out on each other”.	▪ Burnout spectrum ▪ Everyone’s on edge ▪ Staffing difficulty, overwork ▪ Management issues
▪ Impaired job performance	“burn out led to short tempers and poor working relationships”.	▪ Burnout spectrum ▪ Everyone’s on edge
▪ Increased work effort ▪ Diminished quality of patient care	“everyone was stressed and exhausted from the increased call load due to the pandemic. it was hard to keep up with all of the incoming patients which really took a toll on all of us”.	▪ Burnout spectrum ▪ Everyone’s on edge ▪ Staffing difficulty, overwork
▪ Increased work effort ▪ Impaired job performance ▪ Decreased productivity ▪ Disengagement from job/profession	“Everyone is tired. Tired of repeated protocol changes, additional mandates made our job even harder—wearing a mask and gowns carrying equipment, not being able to breathe during heat wave, sweating, sanitizing trucks. While understandable, it took its toll. While Trucks are out to de con other trucks had to take on additional calls, you had to pick up shifts as others were out with COVID, reprimanded/written up for calling out sick with any other illness. Couldn’t see family members because of the nature of our jobs. Covid hysteria led to more aggressive patients and other health care staff. EMS became toxic. You were told not to care about pay or hours or self sacrifice because “that’s who we are” and if you don’t like it, you’re “not cut out for the job”. Everyone can only Give so much before there’s nothing left to give”.	▪ Burnout spectrum ▪ Vaccine, mask, mandate ▪ Staffing difficulty, overwork ▪ Management issues ▪ Systems issues ▪ Toxic, hostile workplace ▪ Policy, protocol issues
▪ Absenteeism, turnover ▪ Impaired job performance and decreased productivity	“people in ems & public safety in general are all physically mentally & emotionally exhausted after dealing with the pandemic with lack of resources, lack of staffing & lack of support for the patients and the community as a whole”.	▪ Burnout spectrum ▪ Staffing difficulty, overwork ▪ Management issues ▪ Systems issues
▪ Increased work effort, absenteeism, and turnover	“people are tired, over worked, everything is constantly changing. staff is getting sick and there are always openings and shortages we cannot fill”.	▪ Burnout spectrum ▪ Staffing difficulty, overwork ▪ COVID-19 magnification
▪ Increased work effort, absenteeism, and turnover ▪ Impaired job performance	“exhausted after people left, vacancies causing more work, call offs creating more work. employees not having enough time to properly care for equipment on duty due to call volume”.	▪ Burnout spectrum ▪ Staffing difficulty, overwork ▪ Systems issues

## Data Availability

Data supporting the reported results are available by request.

## Supplementary Materials

The following supporting information can be downloaded at: https://www.mdpi.com/article/10.3390/ijerph20105861/s1, Table S1: EMS Clinician Descriptions of Conflict Code Dictionary.
