# Supplementary material for: Conflict in the EMS Workforce: An Analysis of an Open-Ended Survey Question Reveals a Complex Assemblage of Stress, Burnout, and Pandemic-Related Factors Influencing Well-Being
Source: Int J Environ Res Public Health. 2023 May 18;20(10):5861. doi: 10.3390/ijerph20105861 (PMC10218282; doi:10.3390/ijerph20105861)
Supplement: Supplementary file 1 [file ijerph-20-05861-s001.zip › ijerph-2346752-supplementary.pdf]

Table S1. EMS Clinician Descriptions of Workplace Conflict: Code Dictionary.

Codes 1-5

**Everyone's on edge**	**Vaccine, mask, mandate**	**Different beliefs, opinions**	**Staffing difficulties, overwork**	**Burnout spectrum**
Conflicts erupt because people are stressed, irritated, frustrated, easily lose patience/become angry	Conflicts erupt because of specific vaccine/mask mandates or unspecified mandates. This code does not include mandatory overtime, which was included with the "staffing difficulties, overwork" code.	Conflicts erupt over differences in beliefs/opinions	Conflicts erupt because of a variety of issues related to staffing (e.g., short staffed, people quit) and/or being overworked for a variety of reasons (e.g., increased patient volume, mandatory overtime)	Conflicts erupt because people are physically, mentally, and emotionally drained. This energy ebb is on a spectrum from being tired/exhausted to being extremely "burned out"
**43 word units:**	**4 word units:**	**28 word units:**	**47 word units:**	**11 word units:**
fight	vaccine	judged	staff	tired
conflict	mask	polarized	turnover	fatigue
cranky	mandate	controversy	shift cover	exhaust
temper	mandatory	opinion	fewer ppl	burn
snap		belief	having to cover	displeasure
irritab		believe	many sick	weary
disagree		divided	understaff	tiresome
argu		took it serious	people leaving	butnout
yell		range of spectrum	manpower	worn out
angrier		view	short hand	brunt out
attitude		not respecting	professionals left	drained
tens		over kill	work short	

on edge		unnecessary	cover shift	
debate		against it	retirement	
striff		vs	coworkers leaving	
stress		treated as if	short we cannot fill	
bicker		taken against	shift conflict	
friction		split	quit	
losing patience		values	shirt staffed	
agitation		concern	Covid leave	
anger		criticizing	people are leaving	
short fuse		real or not	lost several members	
hot point		issue	losing people	
strain		options	people left	
animosity		who do not	out sick	
heated discussions		not so much	pick up shift	
irritated		standpoints	people quite	
frustrat		differences	work faster	
bitch			late transfers	
taking it out			working to much	
emotions			overtime	
angry			overwork	
edgey			more demand	
aggravat			over work	
trigger			worked to cover	
turn against			work hours	
salty			workload	
annoy			overwhelmed	

pissed			call volume	
unhappiness			more calls	
don't have same patients			call load	
small issues to bother			pick up extra shift	
lively discussion			work harder	
			extra work	
			forced to work	
			too many calls	
			over time	

Codes 5-10

**Politics**	**Management issues**	**Policy, protocol issues**	**Covid magnification**	**Covid denial**
Conflicts erupt because of "politics". "Politics" is explicitly or implicitly stated as the reason for conflict	Conflicts erupt because of management or leadership failures (e.g., unsupportive, unfair treatment, double standard)	Conflicts erupt because of unclear, inconsistent, frequently changing, poorly enforced policies/protocols	Conflicts erupt because pre-existing issues/difficulties are magnified/heightened by the Covid pandemic	Conflict is related to denial that Covid is real (by coworkers, the media, politicians, etc.), which makes it difficult to deal with the problem in a reliable consistent way (e.g., vaccine compliance)
**5 word units:**	**14 word units:**	**8 word units:**	**22 word units:**	**18 word units:**
politic	management	protocol	large issue	deniers
Trump	employer	polic	become large ones	serious
redneck	supervisor	guidelines	lowered threshold	very real
pollitic	leaders	standards	insignificant issues	social conditioning
Fox	corporate	guidance	issues amplified	flu

	administration	modify	everything	doesn't exist
	lack of support	subjective	magnified	isn't real
	manager	consistent	increasing the cumulative	not real
	administrative	confusing	a problem before	fake
	reprimand	rules	covid 19 imploded	really exists
	no real attempt		more difficult	is real
	terminat		heightened	was real
	fir		small issues	19nis real
	disci		small things	so-called science
			another layer	Covid is a joke
			already taxing	that it's real
			overly emotional	Covid exist
			become bigger	science and work
			already stressful	
			bleed over	
			little things	
			not an issue	

Codes 10-15

**Systems issues**	**Lack of trust, dishonesty**	**Rights infringement**	**Toxic, hostile workplace**	**Covid conspiracy**
Conflicts erupt because the systems in place to handle the Covid pandemic are inadequate, increasing the burden on EMS workers (e.g., lack of public health	Conflict is related to a lack of trust in coworkers, the public, or the government. There is a perception that people/entities are dishonest (e.g., coworkers lie about	Conflict is related to being forced to do something (e.g., vaccination, mask) that violates your rights/free will (e.g., freedom of choice about what goes into your body)	Conflicts erupt because the work environment is antagonistic to the point of being toxic/hostile (e.g., bullying, hazing)	Conflicts erupt because of differences between those suspicious of a Covid conspiracy and those who believe it is real

infrastructure leads to abuse of 911 system)	their Covid status/call out sick when really taking a break, the CDC lies about the vaccine)			
**19 word units:**	**15 word units:**	**9 word units:**	**10 word units:**	**4 word units:**
operational	false	autonomy	dumping	manufacurer
brittle medical	people using it	own bod	rude	conspiracy
healthcare system	trust	have a choice	bullying	control the people
call 911 instead	not believe	freedom	hazing	man made
excessively long decon	lost all confidence	rights	trash talk	
misuse of the 911	not tell anyone	forcing people	hostil	
longer response time	call out sick	their body	harassed	
divisin of small tasks	rat out	being forced	toxic	
closed trucks	abuse of sick	left to the individual	unfair	
local resources	work sick		week minded	
ems and er	at greater risk			
public health	false information			
supply chain	simply say exposed			
mandate in WA	different standards			
cdc	different rules			
state and feds				
not equipped				
ems				
cms				
